# Ethyl 4-anilino-2-methyl-5-oxo-1-phenyl-2,5-di­hydro-1*H*-pyrrole-2-carboxyl­ate

**DOI:** 10.1107/S1600536813030390

**Published:** 2013-11-09

**Authors:** Mehmet Akkurt, Shaaban K. Mohamed, Mahmoud A. A. Elremaily, Francisco Santoyo-Gonzalez, Mustafa R. Albayati

**Affiliations:** a Department of Physics, Faculty of Sciences, Erciyes University, 38039 Kayseri, Turkey; b Chemistry and Environmental Division, Manchester Metropolitan University, Manchester M1 5GD, England; c Chemistry Department, Faculty of Science, Minia University, 61519 El-Minia, Egypt; d Chemistry Department, Faculty of Science, Sohag University, 82524 Sohag, Egypt; e Department of Organic Chemistry, Faculty of Science, Institute of Biotechnology, Granada University, Granada E-18071, Spain; f Kirkuk University, College of Science, Department of Chemistry, Kirkuk, Iraq

## Abstract

In the title compound, C_20_H_20_N_2_O_3_, the central 2,5-di­hydro-1*H*-pyrrole ring [r.m.s. deviation = 0.014 (1) Å] is oriented at dihedral angles of 77.81 (6) and 25.33 (6)°, respectively, to the attached phenyl ring and the aniline phenyl ring. An intra­molecular N—H⋯O hydrogen bond occurs. In the crystal, mol­ecules are linked through pairs of N—H⋯O hydrogen bonds, forming inversion dimers with an *R*_2_^2^(10) ring motif. Two weak C—H⋯π inter­actions are also observed.

## Related literature

For the synthesis of pyrrolone compounds, see: Shiraki *et al.* (1996 ▶). For the biological activity of lactams, see: Alvi *et al.* (1998 ▶); Li *et al.* (2002 ▶); Mase *et al.* (1999 ▶); Wiedhopf *et al.* (1973 ▶). For bond-length data, see: Allen *et al.* (1987 ▶). For hydrogen-bond motifs, see: Bernstein *et al.* (1995 ▶).
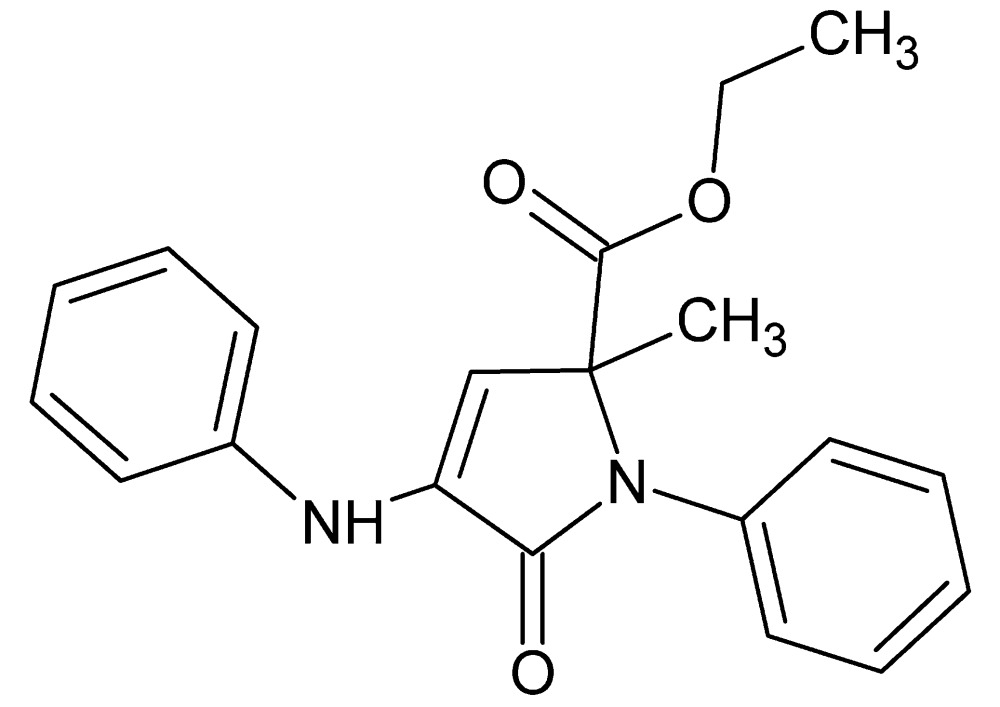


## Experimental

### 

#### Crystal data

C_20_H_20_N_2_O_3_
*M* *_r_* = 336.38
Triclinic, 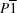

*a* = 5.9071 (6) Å
*b* = 11.3474 (12) Å
*c* = 14.1716 (14) Å
α = 111.467 (2)°
β = 101.113 (3)°
γ = 95.328 (3)°
*V* = 853.45 (15) Å^3^
*Z* = 2
Cu *K*α radiation
μ = 0.72 mm^−1^
*T* = 100 K
0.34 × 0.29 × 0.21 mm

#### Data collection

Bruker APEXII CCD diffractometer
Absorption correction: multi-scan (*SADABS*; Bruker, 2005 ▶) *T*_min_ = 0.783, *T*_max_ = 0.860
18887 measured reflections
3367 independent reflections
3156 reflections with *I* > 2σ(*I*)
*R*_int_ = 0.035

#### Refinement

*R*[*F* ^2^ > 2σ(*F* ^2^)] = 0.037
*wR*(*F* ^2^) = 0.096
*S* = 1.04
3367 reflections
232 parameters
H atoms treated by a mixture of independent and constrained refinement
Δρ_max_ = 0.32 e Å^−3^
Δρ_min_ = −0.17 e Å^−3^

### 

Data collection: *APEX2* (Bruker, 2007 ▶); cell refinement: *SAINT* (Bruker, 2007 ▶); data reduction: *SAINT*; program(s) used to solve structure: *SIR97* (Altomare *et al.*, 1999 ▶); program(s) used to refine structure: *SHELXL97* (Sheldrick, 2008 ▶); molecular graphics: *ORTEP-3 for Windows* (Farrugia, 2012 ▶); software used to prepare material for publication: *WinGX* (Farrugia, 2012 ▶) and *PLATON* (Spek, 2009 ▶).

## Supplementary Material

Crystal structure: contains datablock(s) global, I. DOI: 10.1107/S1600536813030390/is5318sup1.cif

Structure factors: contains datablock(s) I. DOI: 10.1107/S1600536813030390/is5318Isup2.hkl

Supplementary material file. DOI: 10.1107/S1600536813030390/is5318Isup3.cml

Additional supplementary materials:  crystallographic information; 3D view; checkCIF report

## Figures and Tables

**Table 1:** Hydrogen-bond geometry (Å, °) *Cg*2 and *Cg*3 are the centroids of the C8–C13 and C15–C20 phenyl rings, respectively.

*D*—H⋯*A*	*D*—H	H⋯*A*	*D*⋯*A*	*D*—H⋯*A*
N2—H2*N*⋯O3	0.898 (17)	2.443 (16)	2.8247 (14)	105.9 (12)
N2—H2*N*⋯O3^i^	0.898 (17)	2.033 (17)	2.9135 (14)	166.3 (14)
C1—H1*B*⋯*Cg*2^ii^	0.98	2.91	3.6177 (15)	130
C12—H12⋯*Cg*3^iii^	0.95	2.84	3.4865 (14)	126

Symmetry codes: (i) 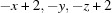; (ii) 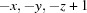; (iii) 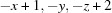.

**Fig. 1.**
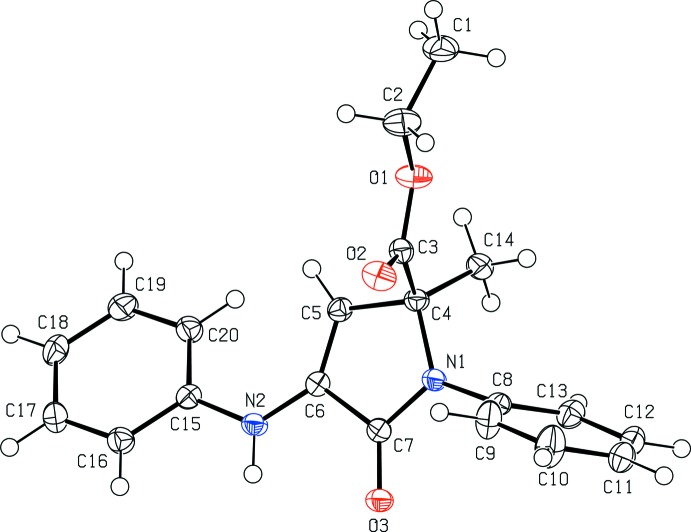
The molecular structure of the title compound with the atom numbering scheme. Displacement ellipsoids for non-H atoms are drawn at the 50% probability level.

**Fig. 2.**
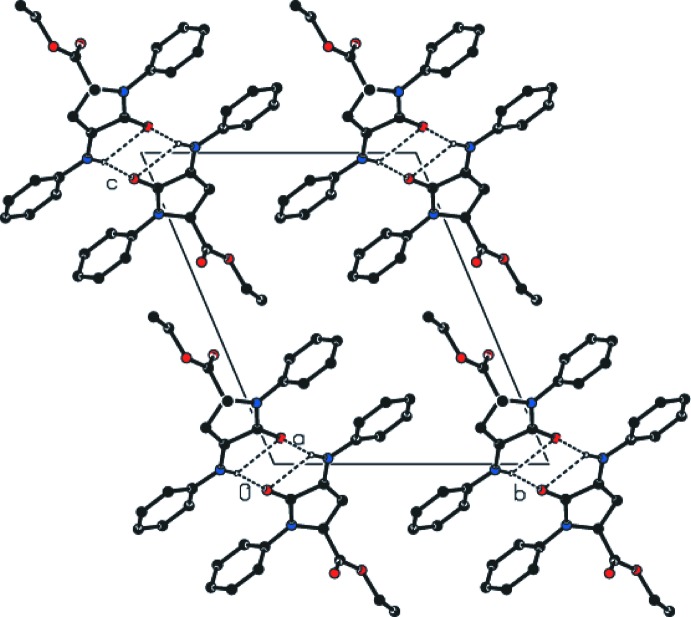
View of the molecular packing and hydrogen bonding (dotted lines) of the title compound along the a axis. H atoms not involved in H bonding are omitted for clarity.

## supplementary crystallographic information

### 1. Comment

Dihydropyrrolone compounds have been reported to display importantant biological activities with better hydrolytic stability. Dihydropyrrolones are known as lactams such as Pulchellalactam, which inhibits CD45 protein, a receptor-like transmembrane protein tyrosine phosphatase, and therefore could be of therapeutic value targeting autoimmune and chronic anti-inflammatory diseases (Alvi *et al.*, 1998; Li *et al.*, 2002). γ-Lactam PI-091 has been reported to display potent activity against platelet aggregation (Shiraki *et al.*, 1996) and jatropham has been proven to be an antitumor alkaloid (Mase *et al.*, 1999; Wiedhopf *et al.*, 1973). Numerous methods to synthesize pyrrol-2-ones have been reported in the literature and the majority of these require multiple steps with low yields. However, one direct conversion strategy has been demonstrated in synthesis of γ-lactam PI-091 (Shiraki *et al.*, 1996). Based on this concept, we herein report the synthesis and crystal structure of the title compound.

In the title compound, the central 2,5-dihydro-1*H*-pyrrole ring (N1/C4–C7) makes dihedral angles of 77.81 (6) and 25.33 (6)° with the two phenyl rings (C8–C13 and C15–C20), respectively (Fig. 1). All bond lengths and bond angles are normal (Allen *et al.*, 1987).

In the crystal structure, pairs of adjacent molecules are linked through intermolecular N—H···O hydrogen bonds (Table 1), forming inversion dimers with *R*^2^_2_(10) ring motifs (Bernstein *et al.*, 1995; Fig. 2). Two weak C—H···π interactions are observed.

### 2. Experimental

In a 50 ml round bottom flask, a mixture of 186 mg of aniline (2 mmol) and 232 mg of ethyl pyruvate (2 mmol) were taken in presence of 8 mol % of Fe_3_O_4_ nanoparticles in 15 ml ethanol/water (*v*/*v*) or glacial acetic acid was stirred well and irradited in microwave for 30 minutes. The progress of the reaction was monitored by TLC. After completion, the solid product was filtered off, washed with water and recrystallized from ethanol. Single crystals suitable for X-ray analysis were obtained by slow evaporation method of an ethanolic solution of the title compound at room temperature.

### 3. Refinement

The C-bound H-atoms were positioned geometrically, with C—H = 0.95, 0.98 and 0.99 Å for aromatic, methyl and methylene H, respectively, and allowed to ride on their respective parent atoms, with *U*_iso_(H) = *xU*_eq_(C), where *x* = 1.5 for methyl H, and *x* = 1.2 for the other H atoms. The N-bound H-atom was located in a difference Fourier map and refined freely.

### Crystal data

**Table:**

C_20_H_20_N_2_O_3_	*Z* = 2
*M**_r_* = 336.38	*F*(000) = 356
Triclinic, *P*1	*D*_x_ = 1.309 Mg m^−^^3^
Hall symbol: -P 1	Cu *K*α radiation, λ = 1.54178 Å
*a* = 5.9071 (6) Å	Cell parameters from 9891 reflections
*b* = 11.3474 (12) Å	θ = 3.5–72.5°
*c* = 14.1716 (14) Å	µ = 0.72 mm^−^^1^
α = 111.467 (2)°	*T* = 100 K
β = 101.113 (3)°	Cubs, colourless
γ = 95.328 (3)°	0.34 × 0.29 × 0.21 mm
*V* = 853.45 (15) Å^3^	

### Data collection

**Table:**

Bruker APEXII CCD diffractometer	3367 independent reflections
Radiation source: sealed tube	3156 reflections with *I* > 2σ(*I*)
Graphite monochromator	*R*_int_ = 0.035
φ and ω scans	θ_max_ = 72.6°, θ_min_ = 3.5°
Absorption correction: multi-scan (*SADABS*; Bruker, 2005)	*h* = −7→7
*T*_min_ = 0.783, *T*_max_ = 0.860	*k* = −14→14
18887 measured reflections	*l* = −17→17

### Refinement

**Table:**

Refinement on *F*^2^	0 restraints
Least-squares matrix: full	H atoms treated by a mixture of independent and constrained refinement
*R*[*F*^2^ > 2σ(*F*^2^)] = 0.037	*w* = 1/[σ^2^(*F*_o_^2^) + (0.0487*P*)^2^ + 0.3108*P*] WHERE *P* = (*F*_o_^2^ + 2*F*_c_^2^)/3
*wR*(*F*^2^) = 0.096	(Δ/σ)_max_ < 0.001
*S* = 1.04	Δρ_max_ = 0.32 e Å^−^^3^
3367 reflections	Δρ_min_ = −0.17 e Å^−^^3^
232 parameters	

### Special details

**Table:**

Geometry. Bond distances, angles *etc*. have been calculated using the rounded fractional coordinates. All su's are estimated from the variances of the (full) variance-covariance matrix. The cell e.s.d.'s are taken into account in the estimation of distances, angles and torsion angles
Refinement. Refinement on *F*^2^ for ALL reflections except those flagged by the user for potential systematic errors. Weighted *R*-factors *wR* and all goodnesses of fit *S* are based on *F*^2^, conventional *R*-factors *R* are based on *F*, with *F* set to zero for negative *F*^2^. The observed criterion of *F*^2^ > σ(*F*^2^) is used only for calculating -*R*-factor-obs *etc*. and is not relevant to the choice of reflections for refinement. *R*-factors based on *F*^2^ are statistically about twice as large as those based on *F*, and *R*-factors based on ALL data will be even larger.

### Fractional atomic coordinates and isotropic or equivalent isotropic displacement parameters (Å^2^)

**Table:**

	*x*	*y*	*z*	*U*_iso_*/*U*_eq_	
O1	0.25546 (15)	0.15386 (9)	0.65884 (6)	0.0278 (3)	
O2	0.56211 (15)	0.05034 (9)	0.64918 (7)	0.0284 (3)	
O3	0.76449 (14)	−0.06561 (7)	0.91641 (6)	0.0245 (2)	
N1	0.46752 (16)	−0.03089 (9)	0.80378 (7)	0.0188 (2)	
N2	0.88879 (16)	0.20496 (9)	1.01844 (7)	0.0198 (3)	
C1	0.0420 (2)	0.20985 (14)	0.52705 (10)	0.0347 (4)	
C2	0.2691 (2)	0.17212 (13)	0.56318 (10)	0.0321 (4)	
C3	0.41016 (19)	0.09001 (10)	0.69110 (8)	0.0202 (3)	
C4	0.37187 (18)	0.07904 (10)	0.79249 (8)	0.0185 (3)	
C5	0.53286 (19)	0.19157 (10)	0.88320 (8)	0.0186 (3)	
C6	0.69734 (18)	0.14945 (10)	0.93602 (8)	0.0174 (3)	
C7	0.65363 (18)	0.00576 (10)	0.88708 (8)	0.0179 (3)	
C8	0.38731 (19)	−0.16263 (10)	0.73392 (8)	0.0195 (3)	
C9	0.5140 (2)	−0.22001 (12)	0.66212 (11)	0.0326 (4)	
C10	0.4452 (2)	−0.34902 (13)	0.59739 (12)	0.0386 (4)	
C11	0.2505 (2)	−0.42071 (11)	0.60400 (10)	0.0279 (3)	
C12	0.1241 (2)	−0.36303 (11)	0.67556 (9)	0.0262 (3)	
C13	0.1920 (2)	−0.23334 (11)	0.74107 (9)	0.0241 (3)	
C14	0.11531 (19)	0.07087 (11)	0.79771 (9)	0.0235 (3)	
C15	0.97223 (19)	0.33611 (10)	1.07830 (8)	0.0189 (3)	
C16	1.2078 (2)	0.37409 (11)	1.13371 (9)	0.0229 (3)	
C17	1.2957 (2)	0.50213 (12)	1.19811 (10)	0.0258 (3)	
C18	1.1522 (2)	0.59446 (11)	1.20871 (10)	0.0263 (3)	
C19	0.9195 (2)	0.55647 (11)	1.15397 (10)	0.0273 (3)	
C20	0.8280 (2)	0.42848 (11)	1.08897 (9)	0.0233 (3)	
H1A	−0.08820	0.14030	0.51190	0.0520*	
H1B	0.04340	0.22560	0.46360	0.0520*	
H1C	0.02220	0.28840	0.58200	0.0520*	
H2A	0.28960	0.09150	0.50930	0.0390*	
H2B	0.40280	0.24060	0.57730	0.0390*	
H2N	0.981 (3)	0.1504 (14)	1.0297 (11)	0.025 (3)*	
H5	0.51940	0.27930	0.90030	0.0220*	
H9	0.64740	−0.17100	0.65730	0.0390*	
H10	0.53200	−0.38860	0.54820	0.0460*	
H11	0.20380	−0.50930	0.55940	0.0340*	
H12	−0.00960	−0.41220	0.68000	0.0310*	
H13	0.10520	−0.19370	0.79020	0.0290*	
H14A	0.01820	0.00030	0.73480	0.0350*	
H14B	0.06470	0.15210	0.80210	0.0350*	
H14C	0.09850	0.05490	0.85970	0.0350*	
H16	1.30760	0.31200	1.12720	0.0270*	
H17	1.45580	0.52720	1.23550	0.0310*	
H18	1.21320	0.68220	1.25280	0.0320*	
H19	0.82040	0.61890	1.16090	0.0330*	
H20	0.66770	0.40400	1.05190	0.0280*	

### Atomic displacement parameters (Å^2^)

**Table:**

	*U*^11^	*U*^22^	*U*^33^	*U*^12^	*U*^13^	*U*^23^
O1	0.0344 (5)	0.0343 (5)	0.0224 (4)	0.0158 (4)	0.0085 (4)	0.0168 (4)
O2	0.0282 (4)	0.0362 (5)	0.0246 (4)	0.0117 (4)	0.0100 (3)	0.0131 (4)
O3	0.0252 (4)	0.0189 (4)	0.0263 (4)	0.0053 (3)	−0.0017 (3)	0.0091 (3)
N1	0.0187 (4)	0.0161 (4)	0.0194 (4)	0.0038 (3)	0.0006 (4)	0.0064 (4)
N2	0.0209 (5)	0.0171 (4)	0.0189 (5)	0.0058 (4)	0.0009 (4)	0.0057 (4)
C1	0.0423 (8)	0.0358 (7)	0.0282 (6)	0.0081 (6)	0.0000 (6)	0.0190 (6)
C2	0.0427 (7)	0.0380 (7)	0.0222 (6)	0.0129 (6)	0.0085 (5)	0.0176 (5)
C3	0.0206 (5)	0.0180 (5)	0.0183 (5)	0.0022 (4)	0.0009 (4)	0.0053 (4)
C4	0.0191 (5)	0.0172 (5)	0.0192 (5)	0.0051 (4)	0.0031 (4)	0.0074 (4)
C5	0.0210 (5)	0.0162 (5)	0.0178 (5)	0.0041 (4)	0.0048 (4)	0.0057 (4)
C6	0.0188 (5)	0.0170 (5)	0.0165 (5)	0.0036 (4)	0.0059 (4)	0.0057 (4)
C7	0.0177 (5)	0.0184 (5)	0.0178 (5)	0.0038 (4)	0.0048 (4)	0.0069 (4)
C8	0.0199 (5)	0.0171 (5)	0.0191 (5)	0.0033 (4)	−0.0004 (4)	0.0069 (4)
C9	0.0248 (6)	0.0221 (6)	0.0442 (8)	0.0011 (5)	0.0142 (5)	0.0034 (5)
C10	0.0354 (7)	0.0237 (6)	0.0481 (8)	0.0039 (5)	0.0205 (6)	−0.0003 (6)
C11	0.0303 (6)	0.0177 (5)	0.0285 (6)	0.0010 (5)	0.0018 (5)	0.0043 (5)
C12	0.0291 (6)	0.0229 (6)	0.0245 (6)	−0.0031 (5)	0.0035 (5)	0.0104 (5)
C13	0.0298 (6)	0.0232 (6)	0.0197 (5)	0.0022 (5)	0.0065 (5)	0.0092 (5)
C14	0.0189 (5)	0.0255 (6)	0.0277 (6)	0.0056 (4)	0.0056 (4)	0.0121 (5)
C15	0.0225 (5)	0.0175 (5)	0.0166 (5)	0.0032 (4)	0.0052 (4)	0.0066 (4)
C16	0.0217 (5)	0.0221 (6)	0.0242 (6)	0.0055 (4)	0.0051 (4)	0.0081 (5)
C17	0.0211 (5)	0.0247 (6)	0.0275 (6)	−0.0004 (4)	0.0036 (5)	0.0080 (5)
C18	0.0302 (6)	0.0174 (5)	0.0271 (6)	−0.0003 (5)	0.0055 (5)	0.0059 (5)
C19	0.0302 (6)	0.0191 (6)	0.0306 (6)	0.0077 (5)	0.0056 (5)	0.0076 (5)
C20	0.0219 (5)	0.0207 (6)	0.0238 (6)	0.0048 (4)	0.0024 (4)	0.0063 (5)

### Geometric parameters (Å, º)

**Table:**

O1—C2	1.4601 (16)	C16—C17	1.3857 (19)
O1—C3	1.3317 (15)	C17—C18	1.3920 (19)
O2—C3	1.2025 (15)	C18—C19	1.3825 (18)
O3—C7	1.2237 (14)	C19—C20	1.3904 (18)
N1—C4	1.4639 (16)	C1—H1A	0.9800
N1—C7	1.3530 (14)	C1—H1B	0.9800
N1—C8	1.4296 (15)	C1—H1C	0.9800
N2—C6	1.3638 (14)	C2—H2A	0.9900
N2—C15	1.3985 (16)	C2—H2B	0.9900
N2—H2N	0.898 (17)	C5—H5	0.9500
C1—C2	1.4991 (19)	C9—H9	0.9500
C3—C4	1.5434 (15)	C10—H10	0.9500
C4—C5	1.5130 (16)	C11—H11	0.9500
C4—C14	1.5274 (16)	C12—H12	0.9500
C5—C6	1.3418 (16)	C13—H13	0.9500
C6—C7	1.4938 (17)	C14—H14A	0.9800
C8—C9	1.3851 (17)	C14—H14B	0.9800
C8—C13	1.3848 (17)	C14—H14C	0.9800
C9—C10	1.383 (2)	C16—H16	0.9500
C10—C11	1.3845 (19)	C17—H17	0.9500
C11—C12	1.3825 (17)	C18—H18	0.9500
C12—C13	1.3912 (18)	C19—H19	0.9500
C15—C20	1.3953 (17)	C20—H20	0.9500
C15—C16	1.3994 (16)		
			
C2—O1—C3	116.61 (9)	C2—C1—H1A	110.00
C4—N1—C7	112.22 (10)	C2—C1—H1B	109.00
C4—N1—C8	125.67 (9)	C2—C1—H1C	109.00
C7—N1—C8	122.05 (10)	H1A—C1—H1B	109.00
C6—N2—C15	127.95 (10)	H1A—C1—H1C	109.00
C15—N2—H2N	116.7 (10)	H1B—C1—H1C	109.00
C6—N2—H2N	114.9 (10)	O1—C2—H2A	111.00
O1—C2—C1	106.07 (10)	O1—C2—H2B	111.00
O1—C3—O2	124.92 (11)	C1—C2—H2A	110.00
O2—C3—C4	125.00 (11)	C1—C2—H2B	111.00
O1—C3—C4	110.04 (9)	H2A—C2—H2B	109.00
N1—C4—C14	111.56 (10)	C4—C5—H5	125.00
C3—C4—C5	107.20 (9)	C6—C5—H5	125.00
N1—C4—C5	102.09 (9)	C8—C9—H9	120.00
C5—C4—C14	113.07 (9)	C10—C9—H9	120.00
C3—C4—C14	112.87 (9)	C9—C10—H10	120.00
N1—C4—C3	109.43 (9)	C11—C10—H10	120.00
C4—C5—C6	110.17 (10)	C10—C11—H11	120.00
C5—C6—C7	108.54 (9)	C12—C11—H11	120.00
N2—C6—C5	135.95 (11)	C11—C12—H12	120.00
N2—C6—C7	115.49 (10)	C13—C12—H12	120.00
O3—C7—N1	126.32 (11)	C8—C13—H13	120.00
O3—C7—C6	126.75 (10)	C12—C13—H13	120.00
N1—C7—C6	106.93 (10)	C4—C14—H14A	109.00
N1—C8—C9	118.96 (11)	C4—C14—H14B	109.00
C9—C8—C13	120.50 (11)	C4—C14—H14C	109.00
N1—C8—C13	120.48 (10)	H14A—C14—H14B	110.00
C8—C9—C10	119.68 (12)	H14A—C14—H14C	109.00
C9—C10—C11	120.31 (12)	H14B—C14—H14C	109.00
C10—C11—C12	119.85 (13)	C15—C16—H16	120.00
C11—C12—C13	120.27 (12)	C17—C16—H16	120.00
C8—C13—C12	119.38 (11)	C16—C17—H17	120.00
N2—C15—C16	117.97 (11)	C18—C17—H17	120.00
C16—C15—C20	119.22 (11)	C17—C18—H18	121.00
N2—C15—C20	122.73 (10)	C19—C18—H18	121.00
C15—C16—C17	120.08 (12)	C18—C19—H19	119.00
C16—C17—C18	120.79 (11)	C20—C19—H19	119.00
C17—C18—C19	118.93 (12)	C15—C20—H20	120.00
C18—C19—C20	121.14 (12)	C19—C20—H20	120.00
C15—C20—C19	119.84 (11)		
			
C3—O1—C2—C1	163.94 (11)	O2—C3—C4—N1	−25.34 (16)
C2—O1—C3—O2	2.26 (18)	C3—C4—C5—C6	−113.96 (11)
C2—O1—C3—C4	−179.93 (10)	C14—C4—C5—C6	121.00 (11)
C7—N1—C8—C13	101.78 (13)	N1—C4—C5—C6	1.02 (12)
C7—N1—C4—C5	0.49 (12)	C4—C5—C6—N2	176.18 (12)
C8—N1—C4—C5	−176.44 (10)	C4—C5—C6—C7	−2.01 (13)
C7—N1—C4—C14	−120.55 (10)	C5—C6—C7—O3	−177.72 (11)
C8—N1—C4—C14	62.53 (13)	N2—C6—C7—N1	−176.31 (9)
C4—N1—C8—C9	101.29 (14)	C5—C6—C7—N1	2.30 (12)
C7—N1—C4—C3	113.83 (10)	N2—C6—C7—O3	3.68 (17)
C8—N1—C4—C3	−63.10 (13)	N1—C8—C9—C10	176.91 (12)
C4—N1—C7—C6	−1.65 (12)	C9—C8—C13—C12	0.19 (18)
C4—N1—C7—O3	178.36 (11)	C13—C8—C9—C10	−0.23 (19)
C8—N1—C7—O3	−4.58 (18)	N1—C8—C13—C12	−176.91 (11)
C4—N1—C8—C13	−81.57 (14)	C8—C9—C10—C11	0.1 (2)
C8—N1—C7—C6	175.41 (9)	C9—C10—C11—C12	0.1 (2)
C7—N1—C8—C9	−75.36 (15)	C10—C11—C12—C13	−0.12 (19)
C15—N2—C6—C7	179.61 (10)	C11—C12—C13—C8	−0.02 (19)
C6—N2—C15—C16	−158.37 (11)	N2—C15—C16—C17	−176.86 (11)
C15—N2—C6—C5	1.5 (2)	C20—C15—C16—C17	−0.13 (18)
C6—N2—C15—C20	25.02 (18)	N2—C15—C20—C19	176.70 (11)
O1—C3—C4—C5	−93.17 (11)	C16—C15—C20—C19	0.13 (17)
O1—C3—C4—N1	156.85 (9)	C15—C16—C17—C18	0.00 (19)
O2—C3—C4—C14	−150.20 (12)	C16—C17—C18—C19	0.1 (2)
O1—C3—C4—C14	31.99 (13)	C17—C18—C19—C20	−0.1 (2)
O2—C3—C4—C5	84.64 (14)	C18—C19—C20—C15	0.00 (19)

### Hydrogen-bond geometry (Å, º)

*Cg*2 and *Cg*3 are the centroids of the C8–C13 and C15–C20 phenyl rings, respectively.

**Table:**

*D*—H···*A*	*D*—H	H···*A*	*D*···*A*	*D*—H···*A*
N2—H2*N*···O3	0.898 (17)	2.443 (16)	2.8247 (14)	105.9 (12)
N2—H2*N*···O3^i^	0.898 (17)	2.033 (17)	2.9135 (14)	166.3 (14)
C1—H1*B*···*Cg*2^ii^	0.98	2.91	3.6177 (15)	130
C12—H12···*Cg*3^iii^	0.95	2.84	3.4865 (14)	126

Symmetry codes: (i) −*x*+2, −*y*, −*z*+2; (ii) −*x*, −*y*, −*z*+1; (iii) −*x*+1, −*y*, −*z*+2.
